# Conformational Analysis in 18-Membered Macrolactones Based on Molecular Modeling

**DOI:** 10.5402/2011/594242

**Published:** 2011-04-19

**Authors:** Salah Belaidi, Dalal Harkati

**Affiliations:** Department of chemistry, Faculty of sciences, University of Biskra, BP 145, 07000 Biskra, Algeria

## Abstract

Conformational analysis of 18-ring membered macrolactones has been carried out using molecular mechanics calculations and molecular dynamics. A high conformational flexibility of macrolactones was obtained, and an important stereoselectivity was observed for the complexed macrolides. For 18d macrolactone, which was presented by a most favored conformer with 20.1% without complex, it was populated with 50.1% in presence of Fe(CO)_3_.

## 1. Introduction

Macrolide antibiotics have been the focus of widespread research due to increasing bacterial resistance [1, 2]. There have been significant synthetic and theoretical efforts to generate new core structures to address this challenge [3]. 

Structure elucidation of a large number of obtained molecules [4], shows the existence of two parts. The first one is a macrocyclic system from 12 to 40 links with several asymmetric centers and lactone function; the second is a sugar part. The two main classes of these macrolides are presented by two molecules; the first is erythromycin A which is an active antibiotic against a large number of bacteria, and the second is amphotericin B which presents a strong anti-fungal. 

Still and Galynker [5] have shown that conformational properties of middle and large size (8 to 14 atoms) might induce a diastereoselection phenomenon for the reactions carried out on these compounds. More precisely, macrocycles which have a double bond (C=C, C=O) and correctly situated substitutes adopt privileged conformations. 

Peripheral attack of the reactive by the less hindered face of *π*-system conduct to a higher stereoselective formation of a new asymmetric center. 

Grée et al. [6] have shown also in some cases the possibility of a stereochemical control induced by tricarbonyliron. So, our objective is to verify if this notion can be extended for cycles with large size. 

In this paper, we propose to study the 18-membered *α*,*β*-unsaturated macrocycle in order to determine the most favored conformations and the influence of Fe(CO)_3_ on conformational flexibility of these macrocycles. 

## 2. Computational Procedures

In our study, the main method of calculation, which we have used, is molecular mechanics. This is considered as the most appropriate method for larger molecules [7]. Programs that we have used are based on Allinger force field [8]. 

This method for structure determination includes a quantum mechanical (VESCF) *π*-system calculation in the iterative sequence. They use Metropolis algorithm [9].

We also used the molecular dynamics (HyperChem) for the conformational research, with following options: 1000°K, in vacuo, step size: 0.001 ps, and relaxation time: 0.1 ps. 

These calculations were carried out with two software packages: HyperChem (8.01) [10], for geometry optimization, and conformational search and Chem3D (8.0) [11], for structural representation. 

Then, our objective is to search the favored conformations, on the basis of energy and geometric considerations with statistical calculations using Boltzmann distribution [12].

**Figure 1:**
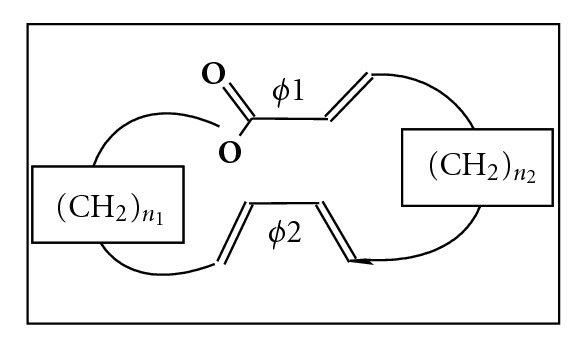
*α*,*β*-unsaturated macrolactone.

In this part of our work, we have undertaken a conformational study of macrocycle 18 (Figure 1), symmetrical which we will design 18s (*n*_1_ = *n*_2_ = 5), dissymmetrical which we will design 18d (*n*_1_ = 4, *n*_2_ = 6), which represent the core group for many antibiotics. 

 We will also try to evaluate the stereoselectivity of addition reactions carried out on functional groups appended to the tricarbonyliron moiety.

## 3. Results and Discussion

The most stable structures can be characterized by three structural characters: the diene group, the *α*,*β*-unsaturated ester group, and the two saturated chains. Thus, we have obtained eight types of conformations which are present in the majority of cases in a 6 kcal/mol energy range above the global minimum. The conformation types are classed from 1 to 8 [13–15]. 

**Figure 2:**
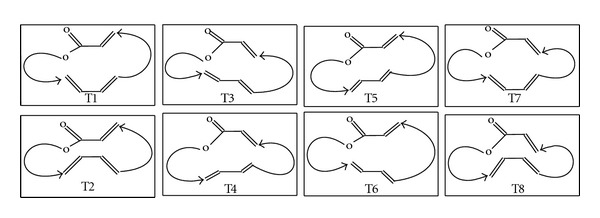
Main conformational types.

For types (2, 4, 6, 8), the two planes of two conformational sites diene and *α*,*β*-unsaturated ester group were pseudoparallels; but for types (1, 3, 5, 7), the two planes of the two sites are pseudoantiparallelsure (Figure 2).

We remark also that for two conformations which we distinguish by the arrangement between the two sites, the dipole moment values are higher for pseudoparallel arrangement and lower in the opposite case (for the macrocycle 18s *μ*(T2) = 2.17 D and *μ*(T1) = 1.97 D).

In 1 kcal/mol difference, the macrocycle 18d is characterized by the first conformer type 6, which is the most favored with 20.1% rate followed by a type 4 with 18.3%. Then, the macrocycle 18s is presented preferably in the type T5 (17.2%) and type T3 (15.0%). 

The percentages of other conformation types are listed in Table 1. The conformer populations of macrocycle 18d are lightly greater than these of macrocycle 18s. For the most favored conformer geometry, the *α*,*β*-unsaturated ester group has s-cis conformation with an angle *ϕ*_1_: O19-C2-C3-C4 = 14.5° for macrocycle 18d and *ϕ*_1_: O19-C2-C3-C4 = 25.0° for cycle 18s. 

The diene group has s-trans conformation with a torsion angle *ϕ*_2_: C11-C12-C13-C14 = 169.4° for 18d and *ϕ*_2_: C10-C11-C12-C13 = 179.5° for 18s. The two systems ester and diene are parallel between themselves. These macrocycles have a very high conformational flexibility. 

However, mobility of dissymmetric macrocycles is lightly less important than that of symmetric macrocycles. 

They present many privileged conformations that do not a priori foresee a diastereoselection for envisaged reactions. This is in agreement with Still's works, on macrocycle 17, which yields many different conformations [16].

We have studied also the exerted effect by tricarbonyliron on conformational flexibility of these macrocycles. We note that organometallic complex can intervene by a very high steric hindrance and also introducing an important rigidification of skeleton. The results of conformational analysis of two complexed macrocycles 18 show that tricarbonyliron has a considerable influence on cycles, because the number of possible conformations was reduced to four types [17–19]. 

In 1 kcal/mol energetic difference, the complexed macrocycle 18s shows three favored conformations and only one favored conformation for 18d. 

The peopling rate of the most stable conformers was increased for complexed macrocycles compared with these without tricarbonyliron (Table 2). 

For macrocycle 18d, which was presented by a preferred conformer with 20.1% without complex, it was populated with 50.1% in presence of Fe(CO)_3_.

We remark also that macrocycles 18s and 18d were presented, respectively, in T1 type with 28.5% and T8 type with 50.1% for the most favored conformers. 

Dienic system was fixed in s-cis conformation for all preferential conformations. The dihedral angle value of dienic system was comprised between 1.0 and 5.4° for 18s cycle and between 4.1 and 13.9° for 18d cycle. The lower deviations of registered dihedral angles compared with normal values were imposed essentially by a cyclic chain [20]. 

The presence of tricarbonyliron motif imposes a minimum steric modifications and introduces an asymmetric element. So, this creates a favorable environment to discrimination between two faces of macrocycles increasing thus a peripheral attack proportion (Figure 4). 

This reasoning was found again in methyl acetates with fluorine containing auxiliaries where intramolecular interaction Li-F creates a steric hindrance around one of two faces causing a diastereofacial selectivity of 94.6 : 5.4 [21]. The lactone function and complexed diene were practically perpendicular at medium plane of the cycle. 

**Figure 3:**
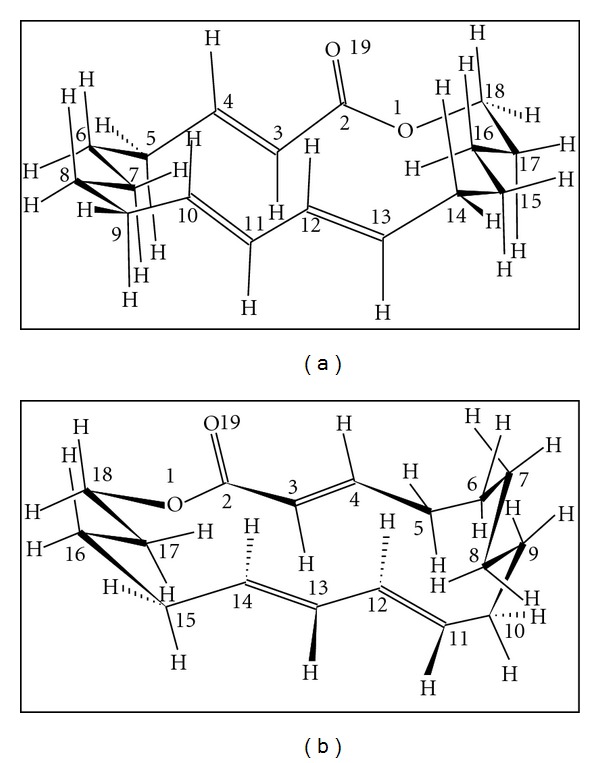
Most favored conformers of macrocycles 18s (a) and 18d (b).

**Figure 4:**
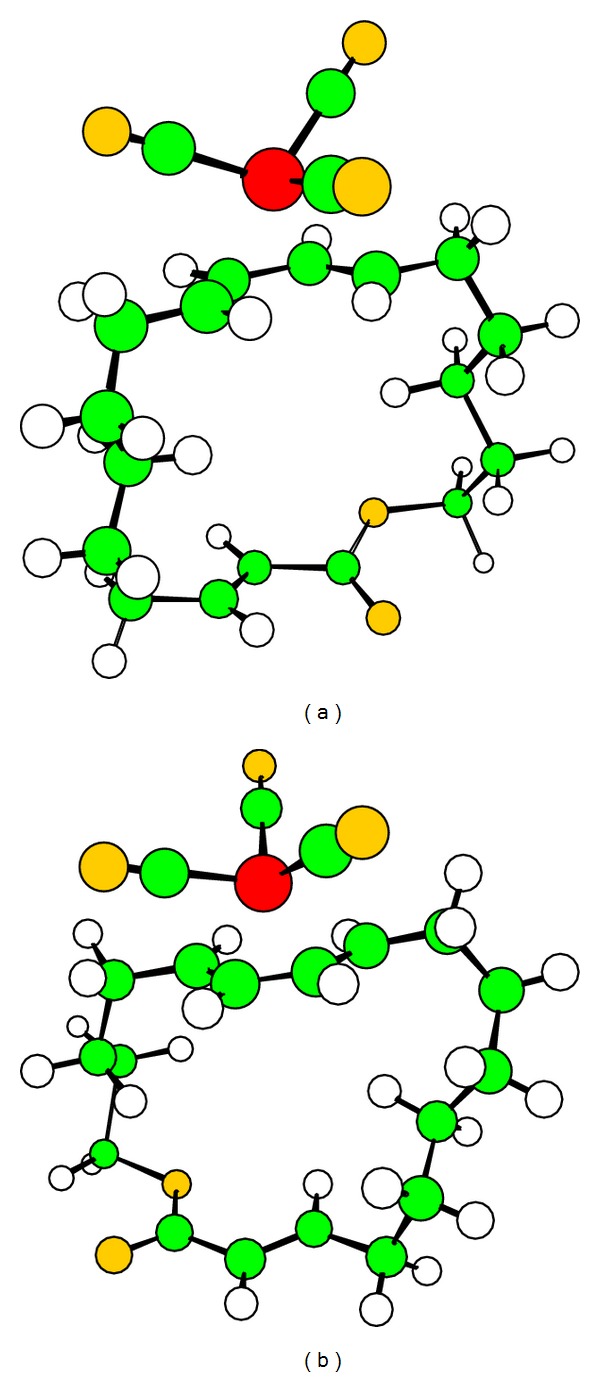
Most favored conformers of complexed macrocycles 18s (a) and 18d (b).

This is in agreement with Still's works that affirm that CH_3_I addition will be executed on the clear face by peripheral attack [5]. The study carried out by Cox and Ley [22], on Fe(CO)_3_ complexes has shown that the presence of complex, which has an important steric effect, induced a diastereoselectivity in addition reactions (Figure 3).

## 4. Conclusion

We conclude that our study shows the existence of a very high conformational flexibility increasing in a majority of noncomplexed macrolactones. 

Finally, the obtained diastereoselectivity for complexed macrolactones is the result of the stereochemical control effect of the tricarbonyliron. It has introduced an asymmetric element, an important steric effect increasing a peripheral attack proportion, and has contributed to the rigidity of the structure. This last factor constitutes a tool of the stereochemical remote control, which permits us to foresee a priori the phenomenon of the stereoselectivity for alkylation reactions.

## Figures and Tables

**Table 1:** Energetic difference and Boltzmann population for different conformationels types.

Macrolactone	18 symmetric (*n*_1_ = *n*_2_ = 5)			18 dissymmetric (*n*_1_ = 4, *n*_2_ = 6)		
	Type	Δ*E*	%	Type	Δ*E*	%
	5	0.00	17.2	6	0.00	20.1
To 1 kcal/mol	3	0.58	15.0	4	0.37	18.3
	4	0.84	14.1	3	0.90	16.1
	6	1.07	13.3	8	1.90	12.6
To 2 kcal/mol	1	1.89	10.9			
	8	1.90	10.9			
	7	2.06	10.5	5	3.03	9.6
Sup to 2 kcal/mol	2	3.05	08.2	1	3.61	8.3
				7	3.66	8.2
				2	4.54	6.7

Δ*E*: Energetic difference to the absolute minimum, %: Boltzmann population.

**Table 2:** Energetic difference and Boltzmann population of different conformational types of complexed macrocycles.

Macrolactone	18 symmetric (*n*_1_ = *n*_2_ = 5)			18 dissymmetric (*n*_1_ = 4, *n*_2_ = 6)		
	Type	Δ*E*	%	Type	Δ*E*	%
	1	0.00	28.5	8	0.00	50.1
to 1 kcal/mol	8	0.30	26.5			
	7	0.54	25.1			
	2	1.47	19.9	7	4.04	18.8
Sup to1 kcal/mol				1	4.30	17.6
				2	5.39	13.5
